# Dextrin Palmitate and Disteardimonium Hectorite Construct a Gel-like EHMC Matrix: Enhanced UVB Photoprotection and Plasma Exposure Modulation

**DOI:** 10.3390/gels12070561

**Published:** 2026-06-23

**Authors:** Zhiwei Li, Yonghang Liang, Chen Liu, Weiyan Wang, Yongliang Li, Zhiyun Du, Li Lin, Junming Zhang, Ling Jiang, Lingna Xie, Meiting Li

**Affiliations:** 1 HBN Research Institute and Biological Laboratory, Shenzhen Hujia Technology (Group) Co., Ltd., Shenzhen 518000, China; lizhiwei@hbn.cn (Z.L.); wangweiyan@hbn.cn (W.W.); 2 Guangdong Engineering Technology Research Center for Functional Skincare Innovation, Shenzhen Hujia Technology (Group) Co., Ltd., Shenzhen 518000, China; 3 School of Biomedical and Pharmaceutical Sciences, Guangdong University of Technology, Guangzhou 511400, China; liangyonghang@mails.gdut.edu.cn (Y.L.); echolau300@gmail.com (C.L.); yongliangli@gdut.edu.cn (Y.L.); zhiyundu@gdut.edu.cn (Z.D.); 2112412120@mail2.gdut.edu.cn (J.Z.); 2112112037@mail2.gdut.edu.cn (L.J.); 4 Foshan Allan Conney Biotechnology Co., Ltd., Foshan 523281, China; annie.lin@allan-conney.com

**Keywords:** 2-ethylhexyl-4-methoxycinnamate, dextrin palmitate, disteardimonium hectorite, gel-like matrix, skin distribution, plasma exposure, exposure modulation

## Abstract

2-Ethylhexyl-4-methoxycinnamate (EHMC) is among the most widely adopted organic UVB filters in commercial sunscreens. Nevertheless, its practical application potential is limited by unfavorable formulation compatibility and safety risks stemming from systemic exposure after topical administration. In this study, an oil-continuous structured gel matrix consisting of EHMC, disteardimonium hectorite (DDH) and dextrin palmitate (DP) was constructed to enhance UVB photoprotection and modulate the plasma exposure profile of EHMC following topical application. Comprehensive characterizations including rheology, XRD, Raman spectroscopy, FTIR spectroscopy, TGA and SEM collectively revealed that the combined incorporation of DDH and DP facilitates matrix structural rearrangement, enables EHMC to bind within the structured network, and promotes the formation of more intact continuous surface films. In vitro SPF assays demonstrated that the finished topical formulation SC-4 delivered superior UVB blocking efficacy compared with the EHMC-only control SC-1; furthermore, SC-4 exhibited improved short-term physical stability under the preset thermal and centrifugal acceleration test conditions. Follow-up skin safety assessments, mass spectrometry imaging (MSI) and pharmacokinetic assays verified that SC-4 elicited no remarkable acute skin irritation across all experimental conditions. Relative to SC-1, the reference formulation with EHMC as the sole UV filter, SC-4 displayed weaker EHMC-related distribution signals in skin tissues, accompanied by lower early plasma EHMC concentrations and a slightly lower AUC_0–48h_ trend. Collectively, these findings indicate that DDH/DP co-assembly serves as a viable matrix-structuring strategy to modulate EHMC-related skin distribution and early plasma exposure. Further research into UVA blocking performance, photostability, skin retention and transdermal permeation profiles, as well as long-term storage stability, is required to advance the development of broad-spectrum sunscreen formulations built on this novel matrix platform.

## 1. Introduction

Since the first report of the Antarctic ozone hole in 1985 [1], depletion of the Earth’s ozone layer has led to a substantial increase in ultraviolet (UV) radiation reaching the terrestrial surface. Excessive exposure to UV radiation is well recognized as a major cause of skin erythema, inflammation, photoaging, and carcinogenesis, thereby prompting extensive scientific and clinical investigation [2]. To mitigate UV-induced skin damage, a variety of photoprotective strategies have been developed, including sun-protective clothing, physical barriers, and topical sunscreens, among which sunscreens remain the most widely adopted approach [3].

Currently available UV filters are generally classified into inorganic blockers, which reflect or scatter incident UV radiation, and organic absorbers, which dissipate UV energy through molecular excitation [4]. Inorganic UV filters such as titanium dioxide (TiO_2_) and zinc oxide (ZnO) offer durable photoprotection but are often associated with poor cosmetic acceptability due to visible whitening on the skin. In contrast, organic UV filters are increasingly favored because of their high UV absorption efficiency, light sensory properties, and formulation versatility.

2-Ethylhexyl-4-methoxycinnamate (EHMC), also known as octinoxate, is one of the most widely used organic UV-B absorbers in commercial sunscreen products [5]. It is commonly incorporated into daily-use sunscreen formulations owing to its strong UV-B absorption and favorable formulation compatibility [6]. Other structurally related organic UV filters include avobenzone, 4-methylbenzylidene camphor, and oxybenzone [7]. Despite their proven efficacy in preventing photoaging and skin cancer, growing concerns have been raised regarding the safety of organic UV filters [8]. Notably, EHMC has been classified as a potential endocrine-disrupting chemical under the European Union Cosmetics Regulation [9]. Upon UV exposure, the predominant trans-EHMC undergoes photoisomerization to the less stable cis isomer, raising additional concerns regarding photostability and biological safety [10]. Trans-EHMC exhibits genotoxicity at concentrations of 0.5–4 mg/mL, whereas cis-EHMC demonstrates genotoxic effects at concentrations as low as 0.25 mg/mL [11]. Furthermore, EHMC photodegradation products, including 4-methoxybenzaldehyde (4-MBA) and cyclic dimers, have been reported to induce cytotoxicity and reduce cellular viability [12]. Consequently, strategies that can improve surface film formation and modulate the exposure profile of EHMC are of practical importance for the safe and effective application of sunscreen agents.

The sun protection factor (SPF) is commonly used to evaluate the UVB protective efficacy of sunscreen formulations [13], and consumer demand for high-SPF products has increased markedly in recent years [14]. However, SPF alone does not ensure sustained protection, as sweating, sebum secretion, and mechanical friction can weaken sunscreen performance during wear [15]. Therefore, matrix-forming excipients are often introduced to improve UV filter retention and promote uniform surface coverage [16]. Previous studies have shown that film-forming agents, such as PEG-75 lanolin, can enhance SPF without increasing UV filter concentration [17]. Composite film-forming systems based on silicone polymers and functional powders have also been reported to improve both SPF and UVA protection factor (UVA-PF) by reducing particle aggregation and improving film uniformity [18]. In addition, crosslinked PDMS coatings can enhance UV protection while reducing the amount of sunscreen agent required [19]. These findings suggest that structurally organized topical matrices may help improve sunscreen efficacy and UV filter retention on the skin surface.

While numerous studies have demonstrated improved SPF performance through the incorporation of film-forming agents, relatively few investigations have examined whether matrix structuring can simultaneously improve UVB photoprotective performance and modulate the skin distribution or systemic exposure of organic UV filters after topical application. For EHMC in particular, existing approaches—including nanoparticulate carriers and surfactant-based delivery systems—have shown promise in reducing transdermal absorption while maintaining UV-blocking efficacy [20,21]. Dextrin-based materials, such as β-cyclodextrin derivatives, have also been explored as film-forming or encapsulating agents capable of improving EHMC photostability and limiting undesirable interactions with formulation components [22,23]. Although dextrin palmitate has been reported in patents to enhance sunscreen SPF, its role in regulating EHMC skin distribution remains insufficiently characterized. Similarly, while disteardimonium hectorite has been shown to enhance the photoprotective performance of organic UV filters, its influence on EHMC skin distribution and plasma exposure after topical application has not been systematically investigated.

Accordingly, the present study aimed to evaluate the role of disteardimonium hectorite and dextrin palmitate in constructing a structured EHMC-containing gel-like matrix. The study focused on three aspects: matrix formation, in vitro UVB photoprotective performance, and EHMC plasma exposure behavior under the tested conditions. By combining material characterization, formulation evaluation, and animal-based safety assessment, this work provides a formulation-level strategy for modulating the distribution and exposure-related behavior of EHMC.

## 2. Results and Discussion

### 2.1. Response Surface Design, Model Analysis, Interaction Analysis and Optimization

Analysis of variance was conducted on the response value drug loading and SPF value fitting model. The significance of each factor and the results of the multivariate regression model analysis of variance are shown in Table 1.

As shown in Table 2, the regression model for response value drug loading exhibits *p* < 0.0001, indicating excellent regression performance and high statistical significance. Meanwhile, the model’s non-significant term yields *p* = 0.1540 > 0.05, suggesting minimal influence of non-experimental factors on the data results. The linear terms A and B, the interaction term BC, and all quadratic terms exerted extremely significant effects (*p* < 0.01) on the response value’s drug loading. The linear term C and the interaction term AC exerted significant effects (*p* < 0.05). The order of influence on the drug loading was: EHMC (A) > DDH (B) > Dextrin Palmitate (C). The regression model for the response value SPF showed *p* < 0.0001, indicating that the fitted equation has good regression performance and extremely high significance. The non-fit term *p* = 0.0801 > 0.05, indicating no outliers in the data. The linear terms A and C, the interaction term AC, and all quadratic terms exerted extremely significant effects (*p* < 0.01) on the SPF value. The linear term B and interaction terms AB and BC exerted significant effects (*p* < 0.05) on the SPF value. The order of influence of factors on the SPF value was: EHMC (A) > dextrin palmitate (C) > DDH (B). In summary, while the order of influence varies slightly for coating efficiency and SPF value, EHMC (A) consistently emerged as the most influential factor for both parameters. This observation aligns with the rationale for selecting drug loading and SPF as primary responses: EHMC concentration directly governs the amount of active sunscreen agent present in the matrix, influencing both surface retention and potential systemic absorption, while SPF quantifies the functional photoprotection provided. The interactions of DDH and DP further modulate matrix structure and film formation, affecting both incorporation efficiency and UV protection, thereby validating the selection of these two parameters as key optimization targets.

As shown in Table 3, the regression model data statistics indicate that the regression coefficient R^2^ for the response variable (drug loading) is 0.9910, demonstrating that this experimental model fits the actual experimental data well. The adjusted R^2^ (R^2^_adj_) is 0.9794, indicating that 97.94% of the data can be explained by this model. The predicted R^2^ (R^2^_pre_) is 0.8952, with a difference from the adjusted coefficient R^2^_adj_ less than 0.2. The coefficient of variation (C.V.) is 3.91% < 10%, demonstrating that the regression equation of this model has high reliability and can effectively reflect the true values. The Adequate Precision signal-to-noise ratio (SNR) of 26.0332 exceeds 4, indicating good model-to-experimental value fit. For the response value SPF, the regression coefficient R^2^ = 0.9914, adjusted R^2^_adj_ = 0.9804, predicted R^2^_pre_ = 0.8895, coefficient of variation C.V.% = 3.68%, and Adequate Precision = 24.7846, all falling within acceptable ranges. In summary, the quadratic response surface regression equations for each response variable demonstrate good fitting quality. Therefore, this model can be used to predict and analyze formulation conditions for the DDH-DP drug loading process.

**Figure 1:**
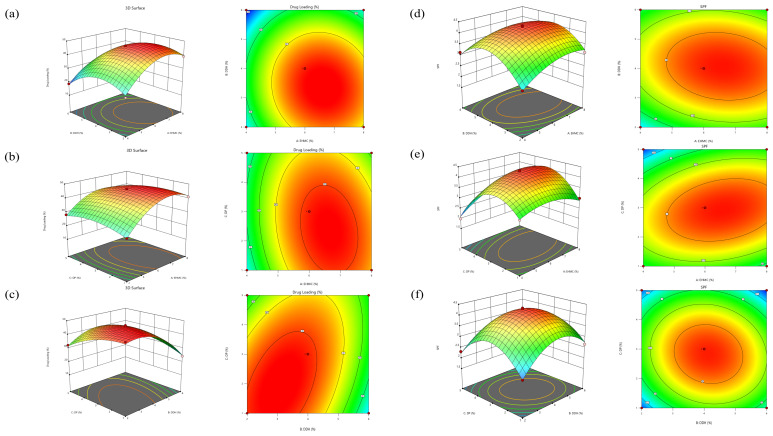
Response surface plots and contour lines of effects of interaction between each factor on Drug loading and SPF. (**a**) 3D response surface and corresponding contour plot showing the interactive effect of EHMC (A, %) and DDH (B, %) on drug loading (%); (**b**) 3D response surface and corresponding contour plot showing the interactive effect of EHMC (A, %) and DP (C, %) on drug loading (%); (**c**) 3D response surface and corresponding contour plot showing the interactive effect of DDH (B, %) and DP (C, %) on drug loading (%); (**d**) 3D response surface and corresponding contour plot showing the interactive effect of EHMC (A, %) and DDH (B, %) on SPF value; (**e**) 3D response surface and corresponding contour plot showing the interactive effect of EHMC (A, %) and DP (C, %) on SPF value; (**f**) 3D response surface and corresponding contour plot showing the interactive effect of DDH (B, %) and DP (C, %) on SPF value.

Based on the ANOVA results of the regression model, Design Expert 13.0 software was used to plot response surface plots and contour plots according to the regression equation. This analysis examined the effects of the interaction between EHMC (A) and DDH (B) on the drug loading and SPF value. The results are represented by contour plots and response surface plots, as shown in Figure 1.

As shown in Figure 1a, the drug loading increases rapidly with rising EHMC (A) levels before plateauing, reaching its maximum at a 7% addition rate. With increasing DDH (B), the drug loading initially rises then declines, exhibiting a slightly gentler trend compared to EHMC (A). This aligns with the single-factor influence patterns of both variables, indicating that EHMC (A) exerts a more pronounced effect during their interaction. The response surface plot exhibits a relatively gentle slope, with sparse contour lines forming a near-circular shape. This indicates moderate interaction between factors and a limited impact on the results, consistent with the ANOVA findings. The contour plot reveals that the drug loading exceeds 40% when EHMC (A) ranges from 5.5% to 8% and DDH (B) ranges from 2% to 5%.

As shown in Figure 1b, the 3D plot of the two-factor interaction between EHMC (A) and DP (C) exhibits steep contours with elliptical isopleths, indicating a high degree of interaction between factors and a significant influence on the results. Combined with the ANOVA results, this interaction reached a significant level (*p* < 0.05). Regarding trend changes, when EHMC (A) is at lower and higher levels, the drug loading exhibits opposite patterns—increasing and decreasing, respectively—as DP (C) increases. Similarly, when DP (C) is at lower and higher levels, the drug loading changes markedly differently as EHMC (A) increases, indicating substantial factor interaction. Contour plots indicate that within the range of EHMC (A): 6–7.5% and DP (C): 1–4%, the predicted drug loading can exceed 45%.

As shown in Figure 1c, when DDH (B) is at lower and higher levels, the drug loading exhibits opposite patterns with increasing DP (C), decreasing and increasing, respectively. When DDH (B) is at a lower level, the rate of decrease in drug loading is more pronounced. Conversely, when DP (C) is at different levels, the pattern of change in drug loading with increasing DDH (B) shows significant differences. These characteristics indicate that changes in either factor level produce divergent trends in outcomes, demonstrating significant interaction between factors with substantial influence on results. Variance analysis confirms this influence reached a highly significant level (P(BC) < 0.01). The response surface plot exhibits steep gradients, while the contour plot forms an elliptical shape, confirming the substantial impact of factor interaction on outcomes. The contour plot reveals that the predicted drug loading is highest when DDH (B) ranges from 2% to 4% and DP (C) ranges from 1% to 3.5%.

As shown in Figure 1d, when DDH (B) is at a low level, the SPF value increases slowly with increasing EHMC (A). When DDH (B) is at a high level, the SPF value first increases and then decreases with increasing EHMC (A), exhibiting a slightly different trend compared to the SPF variation when DDH (B) is at a low level. When EHMC (A) is at a low level, the SPF value rapidly increases and then gradually decreases as DDH (B) increases. When EHMC (A) is at a high level, the SPF value also increases and then decreases as DDH (B) increases, but the rates of increase and decrease are essentially consistent. These patterns indicate distinct SPF responses to changes in both DDH (B) and EHMC (A) levels, reflecting strong interaction between the two factors. The response surface plot exhibits steep gradients with elliptical contour lines. Variance analysis confirms significant interaction (P(AB) < 0.05). The contour plot indicates that when EHMC (A) ranges from 5% to 8% and DDH (B) from 3% to 5%, the SPF value reaches 4 or higher.

As shown in Figure 1e, when DP (C) is at lower and higher levels, the SPF value increases then decreases and gradually increases with increasing EHMC (A), respectively, exhibiting distinct patterns of variation. Similarly, when EHMC (A) is at lower and higher levels, the SPF value exhibits different patterns as DP (C) increases. Considering these patterns and the steepness of the response surface plot, the contour lines form an elliptical shape, indicating that the interaction between the two factors significantly influences the results. The contour lines reveal that when EHMC (A) is between 5% and 8%, and DP (C) is around 2–4%, the predicted SPF value is relatively high.

As shown in Figure 1f, the 3D plot of the interaction between factors DDH (B) and DP (C) is relatively steep, and the contour plot is closer to an ellipse, indicating a high degree of interaction between the factors. This is also consistent with the variance results, where P(BC) < 0.05. The contour plot shows that at the central levels of both factors, the predicted SPF values are relatively high.

In summary, the interactions between factors A and C and between factors B and C significantly influenced drug loading, while the interaction between factors A and B did not significantly affect drug loading. For SPF, the interactions among A–B, A–C, and B–C all showed significant effects. These results indicate that the DDH/DP-assisted matrix formation is governed by multiple component interactions rather than by a single formulation variable.

Using the software’s prediction parameter module, the optimal conditions obtained are: EHMC (A): 6.559%, DDH (B): 4.034%, DP (C): 2.909%. Under these conditions, the predicted drug loading rate Y_1_ is 46.689% and the SPF value Y_2_ is 4.292. Considering practical conditions and operability, the parameters were adjusted to: EHMC (A): 6.5%, DDH (B): 4%, DP (C): 3%.

The practical formulation was further adjusted from the RSM-predicted optimal composition to satisfy comprehensive application performance requirements. Specifically, the EHMC dosage was moderately increased to guarantee sufficient SPF protection, while the DP content was appropriately reduced to maintain matrix stability and avoid excessive system viscosity. The final application formulation was therefore regarded as a practically optimized formulation rather than the mathematical RSM optimum. Its selection was based on a balance among UVB photoprotective performance, viscosity, film-forming behavior, preliminary physical robustness, and application feasibility.

In this study, the selection of drug loading and SPF as dual responses reflected the coordinated objectives of maintaining sufficient UVB photoprotection while improving EHMC in-corporation within the structured matrix. Accordingly, the RSM optimal point was adopted to determine the optimal compositional range that balanced EHMC incorporation and SPF performance, rather than being directly applied as the final practical formulation.

These RSM findings suggest that the performance enhancement of the EHMC@DDH@DP system can be interpreted from the perspective of matrix structuring. The RSM results showed that EHMC concentration exerted the most pronounced influence on drug loading and SPF, which is expected because EHMC is the primary UVB-absorbing component. RSM provided a robust framework for formulation screening, and the highly significant regression models (*p* < 0.0001) confirmed the in-fluence of the selected factors on drug loading and SPF [24,25]. Importantly, the significant interaction terms involving DDH and DP indicated that these excipients did not simply function as conventional additives, but cooperatively regulated matrix organization and photoprotective performance. From a formulation-structure perspective, this behavior is consistent with a gel-like semisolid matrix in which performance is determined not only by the amount of UV filter present, but also by the spatial arrangement and compatibility of the surrounding matrix components around EHMC [26].

### 2.2. Rheological Analysis of the EHMC@DDH@DP Gel-like Matrix

**Figure 2:**
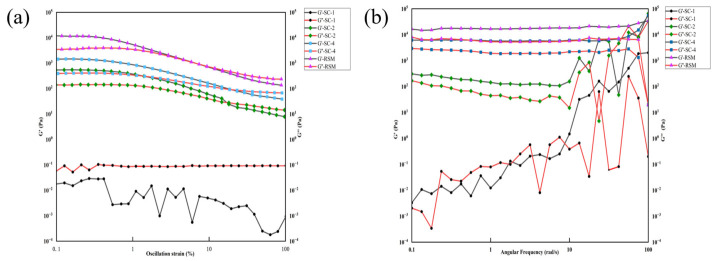
(**a**) Amplitude sweep curves (0.1–100%) of the samples at fixed frequency; (**b**) frequency sweep curves (0.1–100 Hz) of the samples at fixed strain.

Fixed-frequency amplitude sweeps rheological measurements revealed that the viscoelasticity and three-dimensional network structure of the sunscreen system were significantly regulated by the concentrations of DDH, DP and oil-phase EHMC. SC-1 (10% EHMC, without DDH/DP) exhibited no stable linear viscoelastic region, with the storage modulus (G′) consistently lower than the loss modulus (G″), manifesting nearly Newtonian fluid behavior without a three-dimensional gel network, leading to poor static stability and easy delamination. SC-2 (10% EHMC + 4% DDH) formed a weak gel structure with elasticity-dominant behavior at low strain; however, the high EHMC content interfered with the dispersion and crosslinking of hectorite lamellae, resulting in low elastic modulus and narrow linear viscoelastic region with limited structural disturbance resistance. SC-4 (10% EHMC + 4% DDH + 1% DP) achieved synergistic thickening and gelation via DDH-DP cooperation, compensating for the network-weakening effect of EHMC. The elastic modulus plateau of SC-4 was approximately three times that of SC-2, with a broadened linear viscoelastic region and enhanced structural stability. The RSM-optimal formulation (6.5% EHMC + 4% DDH + 3% DP) further reduced EHMC to alleviate network disruption and elevated DP to strengthen hydrophobic association and crosslinking, forming a denser gel-like network. Its elastic modulus plateau was 10 times that of SC-4, with higher static structural stability under the tested conditions and structural rigidity, while retaining favorable shear-thinning behavior for skin spread ability. These results support the formation of a gel-like matrix and provide rheological evidence for cooperative DDH/DP matrix structuring, thereby offering a rheological basis for structural interpretation and formulation optimization (Figure 2a).

Frequency sweep measurements at a fixed strain were performed to further characterize the dynamic viscoelastic behavior and network stability of the formulations. For SC-1 (10% EHMC, no DDH/DP), the storage modulus (G′) was consistently lower than the loss modulus (G″), and both moduli exhibited strong frequency dependence, indicating typical Newtonian fluid behavior without a stable network structure. SC-2 (10% EHMC + 4% DDH) showed weak elasticity dominance at low frequencies but rapid increases in G′ and crossover with G″ at high frequencies, reflecting the frequency sensitivity of the weakly crosslinked physical network with limited structural strength. In contrast, SC-4 (10% EHMC + 4% DDH + 1% DP) exhibited significantly enhanced network stability, with G′ > G″ across the entire frequency range and a distinct elastic plateau at low frequencies, demonstrating reduced frequency dependence and the formation of a stable gel network. The RSM-optimal formulation (6.5% EHMC, 4% DDH, 3% DP) showed typical frequency-independent behavior characteristic of a strong gel, with nearly constant G′ and G″ over a wide frequency range and G′ >> G″. This suggests that the dense three-dimensional crosslinked network exhibits improved dynamic stability, ensuring both static storage stability at low frequencies and structural integrity under high-frequency shear, thus balancing storage performance and application sensory properties (Figure 2b).

### 2.3. Structural Characterization of the EHMC@DDH@DP Gel-like Matrix

**Figure 3:**
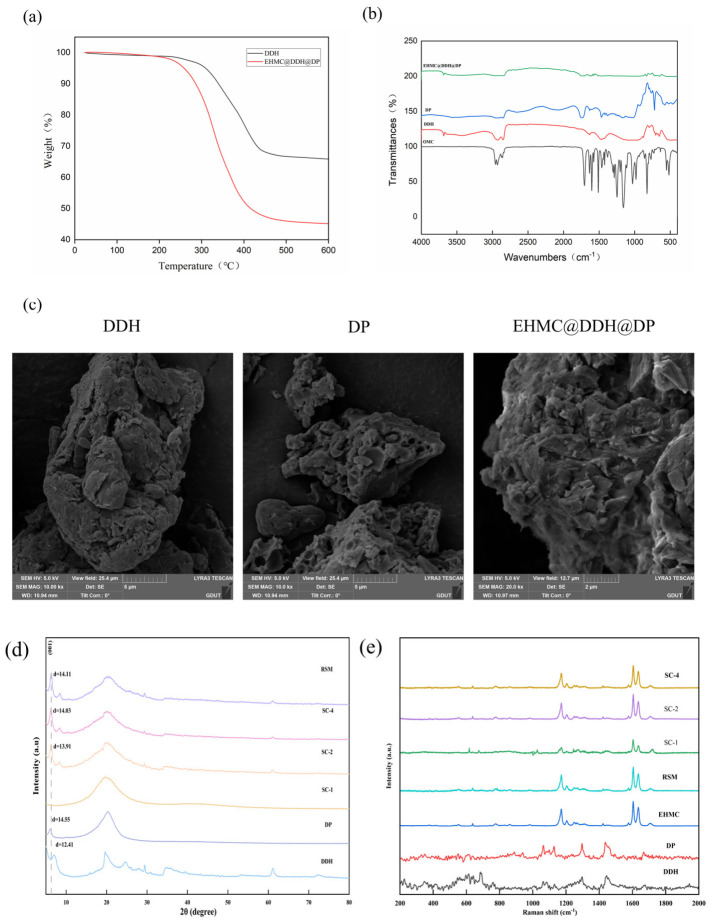
Characterization of the EHMC@DDH@DP gel-like matrix: (**a**) TGA curves of the prepared samples; (**b**) FT-IR spectra of samples with different proportions; (**c**) SEM morphologies of the prepared samples; (**d**) X-ray diffraction of prepared samples; (**e**) Raman spectra of the pre-pared samples.

Thermogravimetric analysis (TG) of pure DDH and the EHMC@DDH@DP gel-like matrix is presented in Figure 3a. The gel-like matrix demonstrates significant mass loss commencing at approximately 200 °C, in contrast to pure DDH, which exhibits notable mass loss at 300 °C. The ultimate residue of the gel-like matrix is approximately 45%, whereas pure DDH retains about 65%. This mass loss phenomenon indicates the successful association of EHMC within the structured matrix, and the earlier initial thermal degradation temperature of the gel-like matrix is mainly attributed to the prior decomposition of the organic components (EHMC and the DP coating).

The FTIR spectra suggest potential intermolecular interactions within the gel-like matrix (Figure 3b). The characteristic C=O stretching vibration of EHMC at ~1720 cm^−1^ was markedly weakened in the EHMC@DDH@DP gel-like matrix compared with its pure form. Simultaneously, the O-H stretching bands of DDH (~3630 cm^−1^ and 3450 cm^−1^) exhibited noticeable changes in shape and position. These observations collectively suggest possible non-covalent interactions, including hydrogen bonding, between EHMC and the matrix-forming components (DDH/DP), which supports the stronger matrix association of EHMC within the structured matrix. Such possible interactions may contribute to altered EHMC molecular mobility within the structured matrix and provide a plausible explanation for the changes in skin distribution and early plasma exposure observed in the matrix-containing formulations.

XRD analysis was further employed to investigate the microstructure of the formulations (Figure 3d). The pure DDH sample exhibited a characteristic diffraction peak of the montmorillonite (001) crystal plane at 2θ ≈ 7.1°. After incorporating EHMC and DP, the (001) characteristic peak of DDH shifted to a lower angle, accompanied by an expanded interlayer spacing. This observation is consistent with the possible hindered movement of EHMC within the layered structure of DDH.

Raman spectroscopy was performed to explore the molecular environment of EHMC in the composite matrix (Figure 3e). The characteristic peaks of EHMC were retained in all composite formulations without obvious shifts or new signal generation, indicating that no chemical reaction occurred between EHMC and the matrix components. Notably, the relative intensity of the EHMC characteristic bands gradually decreased with the introduction of DDH and DP. This change suggests that EHMC molecules may experience restricted molecular mobility within the DDH-DP network, which supports the restricted molecular mobility or stronger matrix association of EHMC within the gel-like matrix.

The structural characterization results further supported the formation of a DDH/DP-assisted gel-like matrix. FTIR showed changes in the EHMC carbonyl band and hydroxyl-related bands of DDH/DP, suggesting possible intermolecular interactions. XRD showed a low-angle shift in the DDH (001) peak after incorporation of EHMC and DP, which is consistent with changes in interlayer spacing. Raman spectra showed reduced EHMC signal intensity in the composite matrices without the appearance of new characteristic peaks, suggesting altered molecular environments and restricted molecular mobility rather than chemical reaction. Together, these results indicate that DDH and DP contributed to matrix reconstruction and possible EHMC association within the structured network. However, these spectroscopic and structural observations should be regarded as supportive rather than definitive mechanistic evidence. Direct proof of molecular confinement or interlayer association would require further quantitative analyses, such as detailed layer-spacing evaluation, molecular simulation, or solid-state structural characterization.

Building on the evidence of intermolecular interactions, the surface morphology of the prepared matrices was characterized by SEM. As shown in Figure 3c, the individual components presented distinct features: DDH as irregular flake-like aggregates and DP with inherent cavities. The EHMC@DDH@DP gel-like matrix, however, showed a markedly different morphology, characterized by surface roughening and the presence of new coating layers. Consistent with the spectroscopic data, this morphological change is consistent with the formation of a structured gel-like matrix, which may contribute to improved surface coverage and favorable matrix association behavior of the sunscreen agent, thereby aligning with the observations from the thermal and FTIR analyses.

The gel-like matrix of the formulations was further characterized by rheological analysis and SEM observation. The linear viscoelastic range and the G′/G″ ratio are consistent with the formation of a relatively stable microstructural network, with DDH and DP concentrations influencing network strength and uniformity. SEM images also appear consistent with a continuous matrix structure, in agreement with the observed rheological behavior. These results offer tentative mechanistic support beyond descriptive observations, suggesting how the composition may influence the gel-like properties of the formulations.

### 2.4. Effect of Matrix Composition on In Vitro UVB Photoprotective Performance

#### 2.4.1. Validation of the RSM-Predicted Formulation

**Figure 4:**
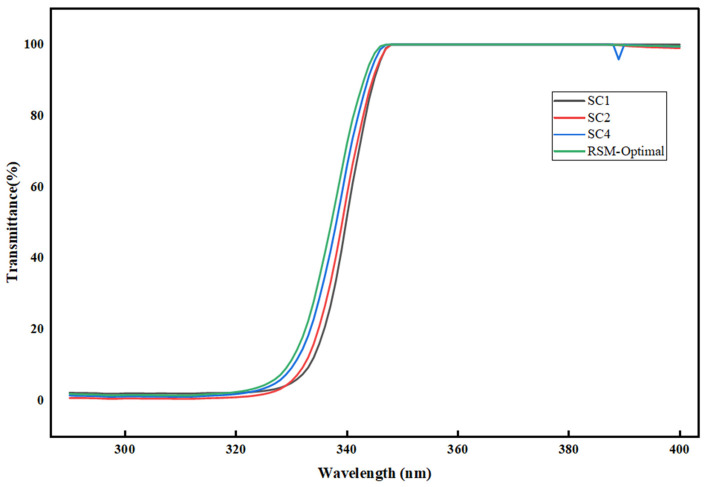
UVA/UVB transmittance of formulations with basic component ratios.

The formulation derived from response surface methodology (RSM) optimization, consisting of 6.5 wt% EHMC, 4 wt% DDH and 3 wt% DP, was experimentally validated to assess its UVB protective performance. The corresponding UV transmittance spectra are presented in Figure 4, and the in vitro SPF results are summarized in Table 4. The RSM-optimized formulation achieved an SPF value of 13.66 ± 0.40, which showed no statistically significant difference compared with the EHMC-only control SC-1 (14.29 ± 0.91, *p* > 0.05). Notably, despite the lower EHMC loading, the RSM-optimal formulation still maintained SPF levels comparable to SC-1. This result demonstrates that the DDH–DP gel network can effectively sustain the intrinsic UVB shielding capability of EHMC, allowing a reasonable reduction in organic sunscreen dosage without compromising basic photoprotective efficacy.

The UV transmittance spectra in Figure 4 further confirmed that the spectral absorption characteristics of all tested formulations were predominantly governed by EHMC, with strong absorption concentrated mainly within the UVB region of 290–320 nm. The light transmittance increased markedly at wavelengths above 320 nm, revealing that the current EHMC-based systems exhibit negligible absorption toward the UVA range. Accordingly, the formulations investigated in this work cannot be regarded as broad-spectrum sunscreen systems due to the lack of effective UVA shielding capacity. Standardized UVA-PF evaluation and the introduction of appropriate UVA-absorbing ingredients will be necessary in subsequent studies to accomplish the development of genuine UVB-oriented sunscreen system sunscreen formulations.

The formulation iteration from RSM theoretical optimum to practical application formulation reflected a necessary transition from pure statistical parameter optimization to product-oriented performance regulation. The RSM predicted optimum (EHMC 6.559%, DP 2.909%) was mathematically solved to balance EHMC retention and SPF response within the designed experimental domain. Nevertheless, practical sunscreen formulation development places greater emphasis on actual photoprotective efficacy as well as comprehensive physicochemical compatibility during application and storage. Appropriately elevating EHMC proportion from 6.5% to 10% complied with the efficacy-oriented design principle of topical sunscreen products, substantially improving actual UVB blocking capacity that could not be fully achieved within the low-concentration range adopted in the RSM design. Meanwhile, moderately decreasing DP dosage from 3% to 1% was conducive to avoiding potential risks associated with excessive addition, such as microstructural heterogeneity, possible crystal separation tendency and undesirable greasy sensory perception. The final application formulation was therefore adjusted on the basis of the RSM-defined composition-performance relationship to better balance UVB protection, viscosity, film formation, and preliminary physical robustness. Accordingly, the finalized application formulation (10% EHMC, 4% DDH, 1% DP) was established on the basis of the component interaction rule revealed by RSM, with the aim of achieving satisfactory photoprotective performance and acceptable overall formulation compatibility. Therefore, SC-4 should be regarded as a practically optimized formulation developed on the basis of the RSM-defined composition-performance relationship, rather than as a direct mathematical optimum.

#### 2.4.2. SPF Performance of the Final Application Formulation

The in vitro SPF values of the prepared formulations were determined to evaluate their UVB photoprotective performance, and the results are presented in Table 5. Although the SPF values of formulations containing DDH and DP (SC-2 and SC-4) were higher than that of the EHMC-only formulation (SC-1), the overall UVB protection efficiency remained moderate even at the relatively high EHMC loading of 10%. These observations indicated that the increase in SPF was relatively limited under the current formulation system, and that higher UV filter content did not lead to a correspondingly substantial improvement in photoprotective efficiency. Notably, the present study only evaluated UVB protection performance (in vitro SPF). UVA protection parameters, including UVAPF and critical wavelength, were not determined; therefore, the broad-spectrum photoprotective capacity of the developed formulations could not be comprehensively assessed.

Within these constraints, comprehensive consideration of photoprotective magnitude and formulation applicability was used to identify SC-4 as the final application formulation. SC-4 provided relatively higher UVB protection while maintaining acceptable physical stability under the tested conditions. This selection prioritized practical protective efficacy on the premise of ensuring basic formulation applicability, rather than simply pursuing the mathematical optimum predicted by the RSM model.

The SPF results also highlight an important formulation principle: UVB protection is not determined solely by UV filter concentration. Although SC-4 improved the in vitro SPF value compared with SC-1, the overall UVB protection efficiency remained moderate considering the 10% EHMC loading. This suggests that the structural organization, film-forming behavior, and optical uniformity of the formulation strongly influence the final photoprotective outcome. Therefore, further studies using a wider range of EHMC concentrations are needed to clarify the relationship between filter loading, matrix structure, and SPF efficiency. It should also be emphasized that the present study focused only on UVB protection. UVA protection performance, including UVAPF and critical wavelength, was not evaluated. Thus, the developed formulations should be regarded as UVB-oriented sunscreen systems at this stage, and further introduction of UVA filters together with systematic broad-spectrum evaluation will be required for future product development.

### 2.5. Effect of Matrix Composition on In Vitro Surface Film Formation

**Figure 5:**
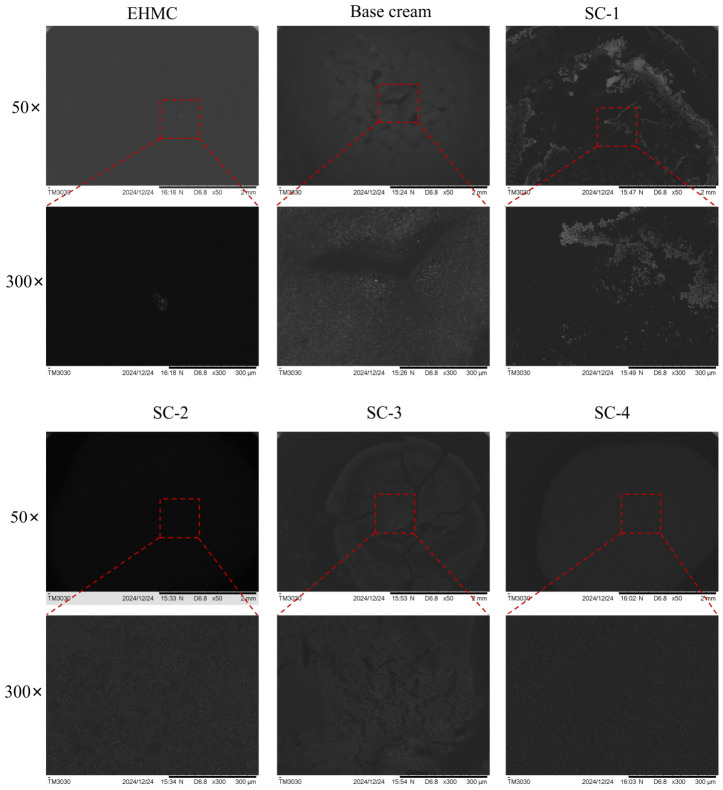
SEM Images of Film Formation for Six Formulations. The red dashed boxes indicate the regions selected for high-magnification observation. The top row presents images at 50× magnification, and the bottom row shows the corresponding magnified views at 300× magnification.

To investigate the effects of different formulation components on micromorphology and in vitro surface film formation property, this study adopted a combined characterization method of optical microscopy (with magnification of 50× and 300×) and scanning electron microscopy (SEM) to systematically observe the microstructural characteristics and surface morphology of EHMC, base cream, and SC series samples (SC-1, SC-2, SC-3, SC-4). As shown in Figure 5, pure EHMC samples exhibited an irregular dot-like cluster distribution; after drying, the SC-1 sample with only EHMC added formed a discontinuous and inhomogeneous non-lamellar film, featuring sharp edges and obvious pores on the material surface; while the introduction of DDH and DP significantly improved the in vitro surface film formation of the samples. Among them, SC-2 and SC-4 formed continuous films with more complete surface structures, and this improved surface coverage was consistent with the SPF results, in which both samples exhibited superior sunscreen performance.

Within this final formulation, DDH likely served as the dominant inorganic structural scaffold, whereas DP acted as an organic binder and interfacial modifier. Their complementary roles contributed to a more coherent gel-like matrix and im-proved surface coverage. Consistent with this interpretation, the in vitro film-formation observations showed that DDH-containing formulations, particularly SC-4, formed more continuous surface films than the porous layer observed in the EHMC-only SC-1 formulation. This improved surface continuity was consistent with the higher in vitro SPF value of SC-4, suggesting that matrix organization and film uniformity contribute to more effective UVB attenuation on the application surface [27,28].

### 2.6. Effect of Matrix Composition on the Stability of Sunscreens

**Figure 6:**
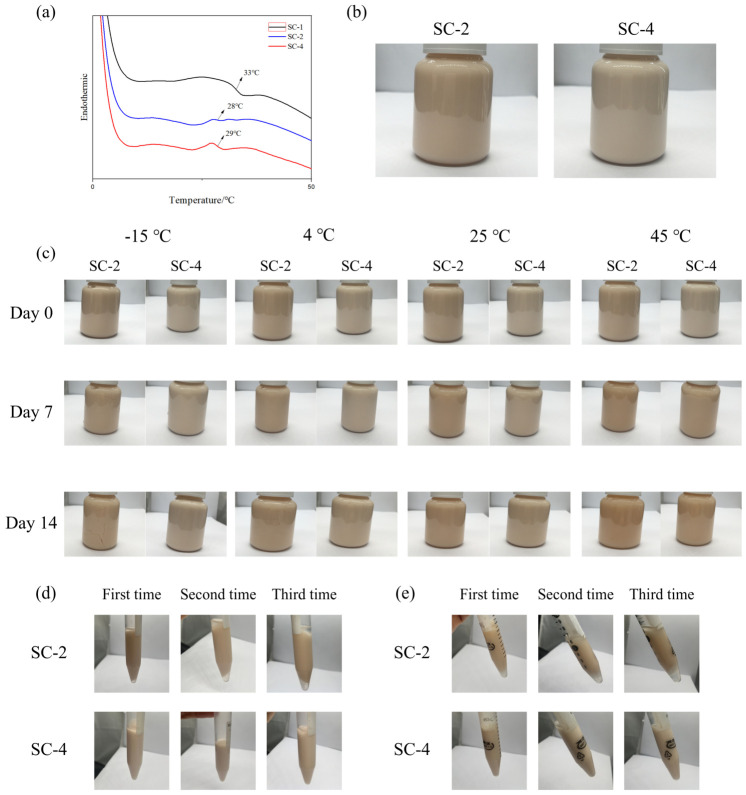
Impact of matrix composition on the stability of sunscreen formulations. (**a**) DSC analysis results. (**b**) Morphological comparison of SC-2 and SC-4 formulations under high-low temperature cycling conditions. (**c**) Morphological changes in SC-2 and SC-4 during temperature stability testing. (**d**) Post-centrifugation morphological comparison of SC-2 and SC-4 samples at 0-day storage. (**e**) Post-centrifugation morphological comparison of SC-2 and SC-4 samples at 7-day storage.

To investigate the thermal behavior characteristics of sunscreen samples with different formulations, this study employed DSC technology to characterize SC-1, SC-2, and SC-4 samples, and systematically analyzed the variation law of their endothermic behavior with temperature. As shown in Figure 6a: the SC-1 sample, which contained only EHMC without the DDH/DP matrix-forming components, exhibited an endothermic process characteristic of glass transition at approximately 30 °C; while the SC-2 sample with DDH alone and the SC-4 sample with both DDH and DP added showed distinct endothermic peaks at 28 °C and 29 °C, respectively. The above results indicate that DDH contributes to matrix flexibility and structural organization through its adsorption and possible interlayer association capacity.

The laser diffraction results indicated that the diluted formulations exhibited broad micron-scale dispersed-domain distributions. Table 6 presents the particle size measurements for Samples SC-2 and SC-4. The D10, D50, and D90 values of SC-2 were 123.2 μm, 160.3 μm, and 207.1 μm, respectively, whereas those of SC-4 were 0.454 μm, 3.894 μm, and 114.8 μm, respectively. SC-2 showed a considerably coarser distribution than SC-4, while SC-4 displayed a much smaller median size but still exhibited a broad upper-tail distribution extending into the micron range. Therefore, these data should be interpreted as apparent particle-size profiles after dilution rather than direct evidence of preliminary physical robustness under tested conditions or uniform dispersion in the original semisolid formulation.

The apparent zeta potential of SC-2 was +9.28 mV, while that of SC-4 was −14.80 mV. Table 7 lists the zeta potential measurements for Samples SC-2 and SC-4. These values were below the commonly accepted |ζ| > 30 mV threshold generally used to indicate sufficient electrostatic stabilization in aqueous dispersions. Accordingly, zeta potential values alone could not support significant electrostatic stabilization in the present formulations. As the tested systems were oil-continuous semisolid preparations, these zeta potential results were only employed as relative indicators reflecting the surface charge features of particles after dilution. It should be emphasized that particle size and zeta potential data were derived from diluted dispersions and provided only supportive information regarding the apparent dispersion state, rather than direct evidence of preliminary physical robustness under tested conditions. In the present work, physical stability was evaluated primarily according to macroscopic appearance, viscosity changes, temperature cycling stability, and centrifugation stability, which served as the key criteria for stability assessment.

The particle size and zeta-potential results should be interpreted cautiously be-cause both measurements were performed after dilution of the original oil-continuous semisolid formulations. Therefore, these values represent apparent dispersion behavior under a unified testing protocol rather than the intrinsic microstructure of the pristine formulation. The zeta-potential values of SC-2 and SC-4 were below the commonly accepted threshold for strong electrostatic stabilization in aqueous dispersions [29], indicating that electrostatic repulsion was unlikely to be the dominant stabilization mechanism.

To systematically evaluate the temperature stability of SC-2 and SC-4 samples, constant-temperature stability tests and high-low temperature cycle stability tests were carried out. The constant-temperature stability test showed that both SC-2 and SC-4 maintained relatively stable appearance at 25 °C (room temperature) and 45 °C for 7 and 14 days. However, both formulations showed reduced stability under low-temperature conditions, and SC-2 exhibited poorer low-temperature tolerance. After 7 days of storage, SC-2 showed oil precipitation and phase separation at both 4 °C and −15 °C, which could be attributed to low temperature-induced oil phase separation. In contrast, SC-4 remained homogeneous and stable at 4 °C, with phase separation observed only at −15 °C, suggesting slightly improved low-temperature tolerance relative to SC-2. This trend was maintained throughout the 14-day storage period.

The viscosity test results showed that the viscosities of both SC-2 and SC-4 increased gradually with prolonged storage, while no obvious changes were observed in skin feel, odor, or appearance color. Table 8 presents the viscosity changes of SC-2 during temperature stability assay. Table 9 shows the viscosity characteristics of SC-4 during temperature stability assay. In addition, after 3 cycles of high-low temperature treatment, no abnormal changes in appearance, viscosity, skin feel, or odor were observed for SC-2 and SC-4, indicating that temperature cycling had no obvious influence on the physicochemical and sensory properties of the two formulations under the tested conditions.

To further assess the physical robustness of the samples, centrifugation tests were performed on fresh samples (0 d) and 7-day stored samples at 3000 rpm for 30 min. After 3 cycles of centrifugation, SC-2 showed significant oil accumulation at the bottom and obvious phase separation, suggesting relatively lower resistance to centrifugal stress. Although SC-4 showed slight oil precipitation after centrifugation, the degree was much milder than that of SC-2, suggesting moderately improved centrifugal stability compared with SC-2. The difference became more obvious after 7 days of storage: SC-2 showed aggravated oil precipitation and phase separation, whereas SC-4 exhibited no marked increase in oil precipitation compared with the initial state. These observations indicate that the incorporation of DP may help enhance the physical stability of the DDH-containing matrix against centrifugation-induced phase separation under the tested accelerated conditions. Under the present test conditions, SC-4 showed better resistance to centrifugation-induced phase separation than SC-2, suggesting that DP may improve the cohesion and physical robustness of the DDH-containing matrix.

It should be noted that all stability evaluations in this study were based on short-term and accelerated tests, which only reflect the physical robustness of the formulations under the tested conditions. Long-term storage stability and accelerated aging studies were not performed in the present work, and further investigation will be required in future research to fully confirm the shelf stability of the developed formulations. Nevertheless, these results should be considered as preliminary physical stability evidence, and long-term storage and accelerated stability studies are still needed to con-firm shelf stability.

### 2.7. Impact of Matrix Composition on Transepidermal Water Loss in Animals

**Figure 7:**
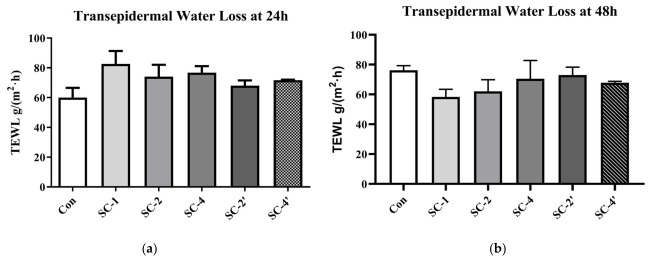
Effect of Film-Forming Conditions on TEWL on Rat Dorsal Skin. (**a**) Comparison of 24 h TEWL values among six prepared sunscreen formulations (including the control group). (**b**) Comparison of 48 h TEWL values among six prepared sunscreen formulations (including the control group).

To evaluate the effect of matrix composition on the skin barrier behavior of sunscreen formulations, this study used the Base cream as the blank control. A Transcutaneous Dehydration Apparatus was employed to detect the Trans epidermal Water Loss (TEWL) of animal epidermis treated with SC-2 and SC-4 sunscreen formulations, and comparative analysis was conducted in combination with the negative control of each group. The results are presented in Figure 7. The test results showed that there was no statistical difference in the TEWL of animal epidermis among the 6 test groups (including the blank control group and test groups) at 24 h and 48 h after treatment. The consistency of this result is presumably related to the fact that all sunscreen formulations are based on an all-oil phase system, which can form a physical barrier on the skin surface to reduce abnormal water loss, thereby weakening the impact of formulation component differences on TEWL. No statistically significant differences in TEWL were detected among the six groups at 24 or 48 h under the present experimental conditions (*p* > 0.05). These results suggest that incorporation of DDH/DP did not measurably impair the rat dorsal skin barrier during the acute observation period. However, the absence of a statistically significant difference should not be interpreted as proof of complete equivalence among formulations.

### 2.8. Impact of Matrix Composition on Skin Irritation in Rat Dorsal Skin

**Figure 8:**
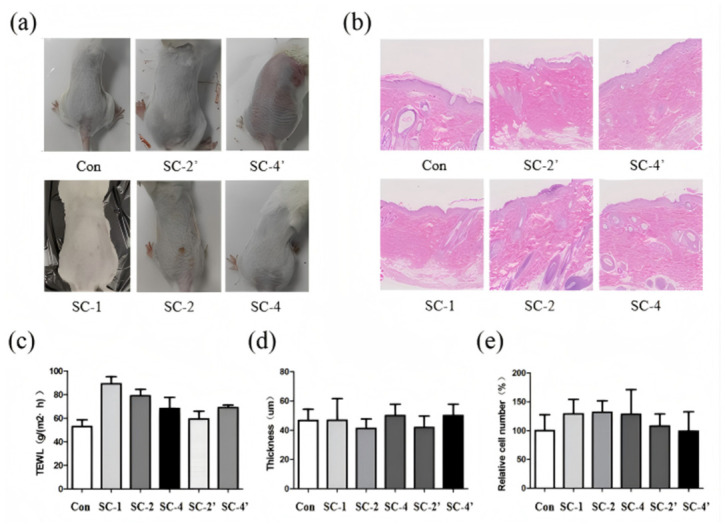
Evaluation of the Irritation Potential of Different Film-Forming Conditions on Rat Dorsal Skin. (**a**) Macroscopic irritation signs induced by six prepared sunscreen formulations (containing the control group) on rat skin. (**b**,**c**) H&E-stained histological sections of rat dorsal skin and comparative analysis of epidermal thickness. (**d**) Quantitative analysis of inflammatory cell infiltration based on H&E staining. (**e**) Quantitative analysis of the relative number of inflammatory cells in rat dorsal skin.

Gross morphological assessment of dorsal skin (Figure 8a) revealed no visible signs of erythema, edema, or irritation in any treatment group throughout the experimental period. This suggests that all formulations, including those containing the DDH/DP matrix components, exhibited low irritancy potential under the tested conditions. Notably, the risk of skin irritation associated with the addition of the film-forming agents appears low under the tested conditions.

H&E-stained dorsal skin sections (Figure 8b,c) revealed that the mean epidermal thickness across all six formulation groups ranged between 40 and 60 μm. Statistical comparison showed no significant differences in epidermal thickness between groups (*p* > 0.05). The absence of epidermal hyperplasia or structural abnormalities further indicated minimal skin irritation. Notably, formulations containing the DDH/DP matrix components did not exhibit enhanced irritancy compared with the EHMC-only comparator (SC-1) or the EHMC-free matched groups (SC-2′ and SC-4′). These findings collectively indicate that the tested sunscreen formulations, even with film-forming additives, caused no significant acute irritation to rat dorsal skin under the experimental conditions. Histological analysis showed no marked increase in epidermal thickness or inflammatory cell infiltration in the SC-4-treated group compared with the corresponding controls.

Inflammatory cell density was quantified from H&E-stained skin histological sections (Figure 8d). No significant intergroup differences were observed at *p* > 0.05. Although formulations containing EHMC exhibited a slight increase in inflammatory cell density compared with the EHMC-free control group, this subtle trend lacked statistical significance. These observations indicate that supplementation with DDH and DP did not aggravate acute skin inflammation under the present experimental conditions. Meanwhile, the mild inflammatory tendency potentially associated with EHMC exposure remains to be further verified in subsequent extended or repeated exposure tests.

The developed matrix also showed favorable preliminary safety-related performance. In the in vivo skin evaluation, no obvious erythema, edema, or visible barrier damage was observed after topical application. These results suggest that the structured DDH/DP-assisted matrix did not cause obvious acute skin irritation under the present experimental conditions [30,31].

### 2.9. Impact of Matrix Composition on EHMC Distribution and Plasma Exposure

**Figure 9:**
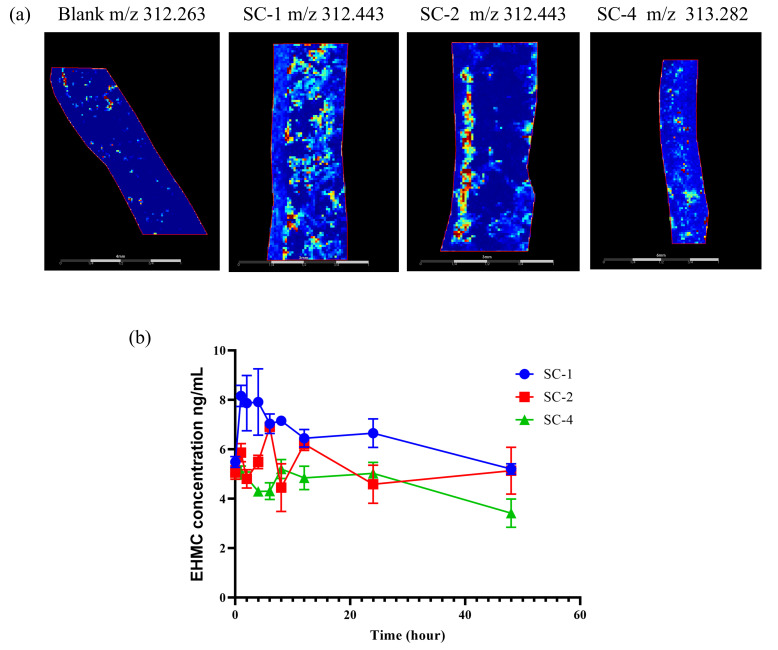
Skin distribution and plasma exposure of EHMC after topical application. (**a**) Mass spectrometry imaging of EHMC distribution in rat dorsal skin. (**b**) Plasma concentration-time profiles of EHMC in rats treated with SC-1, SC-2, and SC-4.

Mass spectrometry imaging was used to visualize the distribution of EHMC in rat dorsal skin. Figure 9 shows the skin distribution and plasma exposure of EHMC after topical application. The blank control group showed weak and diffuse background signals. In contrast, the SC-1 group showed stronger EHMC-related signals in the treated skin area. MSI showed weaker EHMC-related signals in the skin of the SC-4 group than in the EHMC-only SC-1 group, suggesting lower EHMC distribution within skin tissue under the tested conditions. These results suggest that the DDH/DP-containing matrix was associated with lower EHMC distribution in the skin under the tested conditions. However, because surface residue recovery and quantitative skin-layer retention were not assessed, these MSI results should be interpreted as supportive distributional observations rather than direct evidence of enhanced skin-surface retention.

After topical application, EHMC was detectable in plasma samples from all EHMC-containing formulation groups. At 1 h after dosing, the EHMC concentrations were 2.287 ± 0.357, 3.184 ± 1.240, and 2.295 ± 0.540 ng/mL in the SC-1, SC-2, and SC-4 groups, respectively. SC-2 showed the highest early plasma EHMC concentration, whereas SC-4 showed the lowest 1 h concentration.

Table 10 summarizes the pharmacokinetic parameters of EHMC after topical application in rats. The AUC_0–48h_ values were 64.130 ± 10.636, 80.688 ± 38.127, and 60.605 ± 9.405 ng·h/mL for SC-1, SC-2, and SC-4, respectively. Compared with SC-1, the mean AUC_0–48h_ increased by approximately 25.8% in SC-2 and decreased by approximately 5.5% in SC-4. The AUC CV was highest in SC-2, indicating greater inter-individual variability in this group.

The mean concentration-time profiles of SC-1 and SC-2 peaked at 1 h, whereas that of SC-4 peaked at 12 h. However, because individual T_max_ values in SC-4 were dispersed, this result was interpreted as a delayed mean-profile peak trend rather than definitive evidence of sustained release.

The in vivo MSI and pharmacokinetic results provided additional evidence that matrix composition influenced EHMC distribution and systemic exposure behavior after topical application. MSI showed weaker EHMC-related signals in the skin of the SC-4 group than in the EHMC-only SC-1 group, suggesting lower EHMC distribution within skin tissue under the tested conditions. Plasma pharmacokinetic analysis showed that SC-2 had higher early plasma EHMC concentration and higher AUC_0–48h_ than SC-1, indicating that DDH alone did not reduce systemic exposure to EHMC in this formulation system. This phenomenon may be related to formulation-dependent changes in EHMC release, partitioning, or skin surface contact, although the current dataset is not sufficient to determine the exact mechanism.

In contrast, SC-4 showed the lowest 1 h plasma EHMC concentration, a delayed mean-profile peak, and a slightly lower AUC_0–48h_ than SC-1. These findings suggest that the combined DDH/DP matrix may reduce the early plasma appearance of EHMC and slow its mean exposure profile after topical application. However, because the total AUC_0–48h_ reduction was modest and the Cmax of SC-4 was comparable to that of SC-1, these pharmacokinetic data should not be interpreted as definitive evidence of reduced total systemic absorption. Moreover, plasma EHMC exposure is influenced not only by dermal absorption, but also by skin retention, tissue distribution, metabolism, and elimination. Therefore, plasma exposure parameters cannot replace receptor-phase permeation data from Franz diffusion cell studies or direct quantitative skin retention measurements. Future studies using ex vivo human or porcine skin permeation models, skin retention analysis, and in vitro release testing are required to clarify whether the DDH/DP matrix directly modulates EHMC skin permeation or retention.

Taken together, the present findings indicate that DDH/DP co-assembly can im-prove the rheological structure, film-forming behavior, in vitro UVB photoprotective performance, preliminary physical robustness, and exposure-related behavior of EHMC-containing formulations. This provides a formulation-level strategy for coordinating sunscreen efficacy with plasma exposure modulation in organic UV filter systems. Nevertheless, the interpretation of these results remains limited by the experimental model and evaluation scope. The current formulation mainly provides UVB protection, while UVA-PF, in vivo SPF, photostability, and long-term stability were not evaluated. In addition, the rat skin model cannot fully represent human sunscreen use conditions. Further studies using standardized skin retention/permeation models, human or porcine skin, broad-spectrum photoprotection as-says, and long-term stability protocols are required to further validate the applicability of this DDH/DP-assisted matrix platform.

## 3. Conclusions

This study demonstrates that the combined incorporation of DDH and DP enables the construction of a structured oil-continuous gel-like matrix for EHMC and provides a feasible formulation-level strategy for regulating the behavior of organic UV filters in topical sunscreen systems. The DDH/DP-assisted matrix enhanced the elastic-dominant rheological response, promoted more continuous surface film formation, and improved in vitro UVB photoprotective performance without increasing the EHMC content. Compared with the EHMC-only formulation SC-1, the final application formulation SC-4 exhibited preliminary physical robustness in short-term temperature and centrifugation tests and caused no obvious acute skin irritation in the rat model. Moreover, MSI and pharmacokinetic analyses showed that SC-4 was associated with weaker EHMC-related distribution signals in skin tissue, lower early plasma EHMC concentrations, delayed mean-profile peak behavior, and a slightly lower AUC_0–48h_. These findings indicate that DDH/DP co-assembly not only improves the film-forming and UVB-shielding performance of EHMC-containing formulations, but may also modulate EHMC skin distribution and early plasma exposure after topical application. Overall, this work provides experimental evidence that matrix structuring can be used to coordinate photoprotective performance, preliminary physical robustness, and exposure-related behavior of organic sunscreen formulations, thereby offering a promising platform for the development of structured sunscreen systems with improved UVB performance and more favorable exposure-related profiles. Further studies involving standardized skin retention/permeation models, UVA/UVAPF assessment, photostability, and long-term stability are warranted to further validate the applicability of this DDH/DP-assisted matrix platform in broad-spectrum sunscreen development.

## 4. Materials and Methods

### 4.1. Chemicals

2-Ethylhexyl-4-methoxycinnamate (EHMC) and C12-15 alkyl benzoate were purchased from BASF SE (Ludwigshafen am Rhein, Germany). Butylene glycol was obtained from DaiIcel Corporation (Tokyo, Japan). Sodium chloride was purchased from Xilong Scientific Co., Ltd. (Shantou, China). A preservative blend of 90% phenoxyethanol and 10% ethylhexylglycerin (9010) was supplied by Ashland LLC (Norderstedt, Germany). Diisopropyl sebacate was obtained from Stearinerie Dubois Fils S.A. (Boulogne-Billancourt, France). Dextrin palmitate was supplied by Chiba Flour Milling Co., Ltd. (Chiba, Japan). P135 was purchased from Croda Europe Limited (Goole, UK). Cetyl PEG/PPG-10/1 dimethicone was obtained from Evonik Operations GmbH (Wesseling, Germany). Disteardimonium hectorite (DDH) was supplied by Guangzhou Huashi Cosmetics Technology Co., Ltd. (Guangzhou, China). VP/eicosene copolymer was purchased from Ashland Global Holdings Inc. (Wilmington, DC, USA). Cyclopentasiloxane was obtained from Shin-Etsu Chemical Co., Ltd. (Annaka, Japan).

Phosphate-buffered saline (PBS) was purchased from Thermo Fisher Scientific (Suzhou, China). Paraformaldehyde was obtained from Lanjieke Technology Co., Ltd. (Hefei, China). Anhydrous ethanol (≥99.7%, analytical reagent grade) was supplied by Guangdong Guanghua Sci-Tech Co., Ltd. (Shantou, China). Formic acid (≥88%, HPLC grade) and acetonitrile (≥99.9%, HPLC grade) were purchased from Tianjin Comio Chemical Reagent Co., Ltd. (Tianjin, China) and Beijing Marida Technology Co., Ltd. (Beijing, China), respectively. All chemicals were used as received without further purification.

### 4.2. Preparation of the EHMC@DDH@DP Gel-like Matrix

The EHMC@DDH@DP gel-like matrix was constructed using a stepwise assembly strategy. Briefly, EHMC was dissolved in anhydrous ethanol, disteardimonium hectorite (DDH) was dispersed in a 70% (*v*/*v*) ethanol–water solution, and dextrin palmitate (DP) was dissolved in ethyl acetate to obtain individual precursor solutions or suspensions. Subsequently, 5 mL of the EHMC solution was added dropwise to 10 mL of the DDH suspension under vigorous stirring to allow EHMC to associate with the DDH lamellar structure. After equilibration, 5 mL of the DP solution was introduced under continuous stirring to promote the formation of a DP-assisted structured matrix around the EHMC/DDH dispersion. The initial concentrations of EHMC, DDH, and DP were adjusted to achieve the mass ratios specified in Table 11.

### 4.3. Response Surface Methodology

Response surface methodology (RSM) was employed to identify the formulation region in which EHMC loading and in vitro UVB photoprotective performance could be balanced. EHMC concentration (factor A), DDH concentration (factor B), and DP concentration (factor C) were selected as independent variables. The factor levels for the response surface test are listed in Table 12. Drug loading was selected as one response because it reflects the proportion of EHMC incorporated within the structured matrix, which is relevant to matrix association and formulation-level exposure-related behavior. SPF was selected as the second response because it directly reflects the functional UVB photoprotective performance of the formulation. The purpose of the RSM study was therefore to guide formulation design and identify a rational composition space, rather than to define the final application formulation solely by the mathematical optimum. A multivariate experimental design was constructed, and regression analysis was performed to assess the significance of individual factors and their interactions. Analysis of variance (ANOVA) was applied to determine the statistical significance and predictive reliability of the fitted models. The optimal formulation parameters predicted by the RSM model were subsequently adjusted based on practical formulation considerations and experimentally validated.

Using Design Expert 13 software, regression analysis was performed on the response surface test results and response values (drug loading and SPF value) from Table 11. A second-order response surface regression model was employed, yielding the following regression equations for drug loading (Y_1_) (1) and SPF value (Y_2_) (2):

Y_1_ = 45.89 + 5.63A − 5.01B − 1.54C − 1.50AB − 2.25AC + 5.32BC − 9.85A^2^ − 8.69B^2^ − 3.68C^2^ (1)

Y_2_ = 4.26 + 0.22A + 0.10B − 0.16C − 0.13AB + 0.30AC − 0.19BC − 0.37A^2^ − 0.88B^2^ − 1.07C^2^ (2)

### 4.4. Characterization of the EHMC@DDH@DP Gel-like Matrix

#### 4.4.1. Rheological Assay

Rheological properties of the samples were determined using a rotational rheometer (Mars 40, Thermo Fisher, Waltham, MA, USA) with a 60 mm parallel plate under oscillatory mode at 25 °C.

Amplitude sweep tests were performed at a constant frequency of 1 Hz over a strain range of 0.01–100% to obtain strain–modulus curves. The storage modulus (G′) and loss modulus (G″) were recorded to determine the linear viscoelastic region and evaluate gel strength and structural stability.

Frequency sweep tests were carried out at a constant strain of 0.5% (within the linear viscoelastic region) over a frequency range of 0.1–100 Hz. A gel structure was identified by G′ > G″, indicating dominant elastic behavior. A nearly unchanged G′ over the tested frequency range confirmed a stable gel network. In this study, “gel-like” behavior was operationally defined by the presence of an elastic-dominant response (G′ > G″) over the tested frequency range together with a measurable linear viscoelastic region, rather than by the formation of a classical molecular gel or hydrogel.

#### 4.4.2. Thermogravimetric Analysis (TGA)

The thermal stability of the samples was evaluated by thermogravimetric analysis (TGA) using a thermogravimetric analyzer (TGA 4000, Perkin Elmer, Shelton, CT, USA). Approximately 5–10 mg of sample was placed in an alumina crucible and heated from room temperature to 600 °C at a constant rate of 10 °C/min under a nitrogen flow of 50 mL/min, while the mass change was continuously recorded.

#### 4.4.3. Fourier Transform Infrared (FTIR) Spectroscopy

Fourier-transform infrared (Nicolet is50 R, Thermo Fisher, Waltham, MA, USA) spectroscopy was used to explore potential intermolecular interactions among EHMC, DDH, and DP in the structured matrix. All samples were prepared as KBr pellets and analyzed over the wavenumber range of 4000–400 cm^−1^ at a resolution of 4 cm^−1^. Characteristic absorption bands corresponding to EHMC carbonyl stretching and hydroxyl vibrations of DDH/DP were compared between individual components and composite systems to examine changes in molecular environments.

#### 4.4.4. Scanning Electron Microscopy (SEM)

The surface morphology and microstructure of the samples were observed using a scanning electron microscope (Lyra 3 Xmu, Tescan, Brno-Kohoutovice, Czech Republic). Prior to testing, a small amount of powdered sample was uniformly dispersed on conductive adhesive. The sample surface underwent gold sputtering treatment for approximately 60–90 s under high vacuum to enhance conductivity and obtain clear secondary electron images. Subsequently, the sample surface was examined at an appropriate acceleration voltage, and representative regions were photographed.

#### 4.4.5. X-Ray Diffraction Assay

A small amount of each sample was analyzed by X-ray diffraction (D8, Advance, Bruker, Darmstadt, Germany) to obtain the diffraction patterns and crystallinity characteristics. The measurements were performed under the following conditions: scanning range of 3–60° (2θ), scanning speed of 8°/min, operating voltage of 40 kV, and operating current of 30 mA.

#### 4.4.6. Raman Spectroscopy

A small amount of each sample was placed on a glass slide and analyzed using a confocal Raman microscope (Labram Hr Evolution, Horiba Jobin Yvon, Palaiseau, France). Spectra were recorded using an excitation wavelength of 785 nm, a grating of 600 lines/mm, and a 50× long-working-distance objective. The spectra were collected over 400–4000 cm^−1^, with particular attention to the EHMC characteristic bands in the fingerprint region (approximately 1600–1800 cm^−1^). Each measurement was performed with an acquisition time of 10 s and 10–20 accumulations to improve the signal-to-noise ratio.

### 4.5. Preparation of Sunscreen Formulation Samples

#### 4.5.1. Preparation of the Aqueous Phase

According to the sunscreen formulation composition specified in Table 13, the aqueous phase was prepared by sequentially mixing butylene glycol and deionized water under continuous stirring. Sodium chloride was then added and homogenized until complete dissolution, followed by the incorporation of phenoxyethanol/ethylhexylglycerin preservative blend (Euxyl PE 9010; 90% phenoxyethanol and 10% ethylhexylglycerin) under thorough agitation.

#### 4.5.2. Preparation of the Oil Phase

To prepare the oil phase, ethylhexyl methoxycinnamate, diisopropyl sebacate, and dextrin palmitate were heated to 100 °C to ensure complete solubilization. The temperature was then adjusted to 70–80 °C, and P135 together with VP/Eicosene Copolymer was introduced and dissolved under mechanical stirring. Concurrently, a DDH dispersion was prepared by gradually adding DDH into a premixed oil phase containing cetyl PEG/PPG-10/1 dimethicone and C12-15 alkyl benzoate under high-speed dispersion. The dispersion was processed using a high-shear homogenizer (T25, IKA, Staufen, Germany) at 10,000 rpm for 3 min at 80 °C until no visible agglomerates were observed. The prepared DDH dispersion was then added to the oil mixture, followed by the incorporation of cyclopentasiloxane, and the resulting oil phase was stirred until uniform.

#### 4.5.3. Mixing of Aqueous and Oil Phases

The aqueous phase was gradually incorporated into the oil phase. The combined mixture was then homogenized using a high-shear homogenizer (T25, IKA, Staufen, Germany) at 12,000 rpm for 3–5 min to achieve uniform emulsification. The resulting emulsion was cooled to ambient temperature under continuous stirring before final discharge.

### 4.6. Sun Protection Factor Assay

A precisely measured 35.0 mg of each formulation was uniformly applied onto a 50 mm × 50 mm polymethyl methacrylate (PMMA) plate. In vitro SPF was measured using a UV transmittance analyzer (UV-2000S, Labsphere, North Sutton, NH, USA). Measurements were performed at 9 different positions per plate and repeated using independently prepared samples. The reported SPF values are therefore interpreted as in vitro UVB photoprotective indices rather than substitutes for in vivo SPF results. The test procedure was performed in accordance with ISO 24443:2021 [32].

### 4.7. In Vitro Surface Film Formation Assay

To evaluate in vitro surface film formation, pure EHMC, base cream, and SC-series formulations (SC-1, SC-2, SC-3, and SC-4) were uniformly applied onto wide-spaced conductive adhesive substrates. Coatings were prepared under bubble-free conditions and stored in a covered, light-protected container for 48 h to allow natural drying. After drying, the samples were sputter-coated with platinum using an automatic gold/carbon coater (Cressington 108auto, Shanghai, China) and examined using a benchtop scanning electron microscope (TM3030, Hitachi, Tokyo, Japan). Representative images were collected for comparison of film continuity, surface defects, and matrix morphology.

### 4.8. Differential Scanning Calorimetry

A sample weighing 8–10 mg was placed in the sample pan and analyzed using a differential scanning calorimetry (DSC) instrument (DSC 204F1 Phoenix, Netzsch, Selb, Germany). The parameters were set as follows:

(i) AIR (80/20): 250.3 mL/min;
(ii) NITROGEN: 250.0 mL/min;
(iii) Temperature ramp range: 0–100 °C;
(iv) Heating rate: 10 °C/min.

### 4.9. Particle Size Assay

Because the tested samples were oil-continuous semisolid sunscreen formulations, particle size data obtained after dilution were used only as supportive indicators of the apparent dispersion state rather than absolute descriptors of the pristine formulations. Briefly, each sample was pre-diluted with C12-15 alkyl benzoate at a sample-to-diluent ratio of 2:8 (*v*/*v*). The pre-diluted sample was then added dropwise into ultrapure water under continuous stirring at 550 rpm and ultrasonic power of 50 W until the obscuration rate was maintained at 2.0–12.0%. Particle size distribution was measured using a laser diffraction particle size analyzer (Bettersize 2600, Dandong Bettersize Instruments Ltd., Dandong, China; measurement range: 0.1–5000 μm). The optical parameters were set according to the instrument default settings for micron-scale particulate dispersions. Each sample was measured in triplicate, and D10, D50, and D90 values were reported.

### 4.10. Zeta Potential Assay

Zeta potential measurement was performed to evaluate the surface charge characteristics and relative dispersion behaviour of particles within the developed formulations. Consistent with the particle size test rationale, the tested oil-continuous semisolid systems preclude the acquisition of authentic particle electrical parameters via direct testing of undiluted samples. As such, zeta-potential values determined from diluted dispersions served only as auxiliary relative indicators, and could not fully represent the absolute surface electrical properties of particles in the original formulation matrix. All samples were dispersed in phosphate-buffered saline (PBS) following a unified dilution protocol to ensure consistent testing conditions. Zeta potential measurement was conducted using a Nanoparticle Size and Zeta Potential Analyzer (Zetasizer Nano ZS, Malvern Panalytical, Worcestershire, UK). Each sample was measured in triplicate.

### 4.11. Temperature Stability

The formulation samples were stored at −15 °C, 4 °C, 25 °C, and 45 °C for 0, 7, and 14 days. At each time point, the samples were assessed for physical appearance, textural properties, odor, and color, while viscosity was measured to assess rheological stability under varying storage conditions.

### 4.12. Temperature Cycling Stability Assay

The formulation samples were cycled through four temperature conditions (−15 °C, 4 °C, 25 °C, and 45 °C) for three times. Following each cycle, the samples were evaluated for physical appearance, textural properties, odor, and color, with viscosity measurements conducted to assess rheological stability under cyclic thermal stress.

### 4.13. Centrifugation Stability Assay

The formulation samples were placed into 15 mL centrifuge tubes and centrifuged at 3000 rpm for 30 min per cycle, with this process repeated three times consecutively. After each centrifugation cycle, the samples were visually inspected and evaluated for physical characteristics including appearance, phase separation, and structural integrity to assess stability under repeated centrifugal stress.

### 4.14. Animal Study

Female Sprague Dawley rats (4 months old; 250–300 g), purchased from Guangdong Medical Laboratory Animal Center (Guangzhou, China). All procedures involving experimental animals in this study were reviewed and approved by the Animal Ethics Committee of Guangzhou Yongnuo Medical Laboratory Animal Center (Approval Number: IACUC-AEWC-F250107001). Rats were randomly allocated into the following six groups (*n* = 8/group):

(i) Blank control group (CON): base formulation without EHMC, DDH, or DP;
(ii) SC-2 group: formulation containing EHMC and DDH;
(iii) SC-4 group: formulation containing EHMC, DDH, and DP;
(iv) SC-1 group: EHMC-only formulation;
(v) SC-2′ group: SC-2-matched formulation without EHMC;
(vi) SC-4′ group: SC-4-matched formulation without EHMC.

After two days of acclimatization, the dorsal hair of rats was shaved to create a 3 cm × 4 cm area, and depilatory cream was applied. After 2 min, the cream was thoroughly rinsed off with water. The rats were allowed to rest for 24 h with unlimited access to food and water. On the following day, a 2 mg/cm^2^ dose of the test sample was uniformly applied to the depilated area using a gloved fingertip. A gauze barrier was placed over the area to prevent ingestion and potential interference with results, while all rats had free access to food and water.

#### 4.14.1. Blood Collection and Pharmacokinetic Analysis

Blood samples (approximately 0.4–0.5 mL) were collected from the retro-orbital plexus at 0, 1, 2, 4, 6, 8, 12, 24, and 48 h after topical application. The original animal study included eight rats per group. All rats were sampled at 0–12 h. At the 24 h time point, blood was first collected, and four rats per group were then randomly selected and euthanized for skin tissue analysis. Because four rats per group were euthanized at 24 h, only the remaining four rats per group had complete 0–48 h plasma concentration–time profiles. Therefore, the pharmacokinetic exposure analysis was performed descriptively using four complete profiles per EHMC-containing group. All samples were placed on ice prior to processing. Plasma was separated by centrifugation at 800× *g* for 15 min at 4 °C and stored at −80 °C until analysis.

Plasma EHMC concentrations were quantified using LC-MS/MS. Pharmacokinetic parameters, including the area under the concentration–time curve from 0 to 48 h (AUC_0–48h_), maximum plasma concentration (C_max_), and time to reach maximum concentration (T_max_), were calculated by non-compartmental analysis. AUC_0–48h_ was calculated using the linear trapezoidal method. C_max_ was defined as the highest observed plasma concentration, and T_max_ was defined as the corresponding sampling time. These parameters were used as descriptive indicators of systemic EHMC exposure after topical application. No steady-state permeation flux, permeability coefficient, cumulative permeated amount, or lag time was calculated because no Franz diffusion cell permeation experiment was included in the revised analysis.

#### 4.14.2. Gross Morphological Analysis and Transepidermal Water Loss (TEWL) Assay in Rats

At 24 h and 48 h post-application, four rats were randomly selected from each group at the respective time points. The test samples were gently removed from the depilated skin area using sterile cotton swabs. The treated skin was photographed under standardized lighting conditions to document macroscopic morphological changes. Transepidermal water loss (TEWL) was subsequently measured using a Transcutaneous dehydration apparatus (TM Hex, Courage+Khazaka electronic GmbH, Köln, Germany).

#### 4.14.3. Processing of Dorsal Skin Tissue in Rats

At the experimental endpoints of 24 h and 48 h, four rats per group were randomly euthanized. The dorsal skin tissue was carefully excised and divided into two portions:

(i) Fixed in 4% paraformaldehyde (PFA) for 24 h at 4 °C (for subsequent HE staining and histopathological sectioning);
(ii) Flash-frozen in liquid nitrogen and stored at −80 °C (for mass spectrometry imaging (MSI) analysis).

#### 4.14.4. Histological Sectioning, H&E Staining, and Analysis of Dorsal Skin Tissue in Rats

The H&E-stained sections were scanned using a fully automated digital slide scanning system (AxioScan.Z1, ZEISS, Oberkochen, Germany). Epidermal thickness and inflammatory cell density were quantified using ImageJ software (Version 1.8.0 t, National Institutes of Health, Bethesda, MD, USA) following standardized morphometric protocols.

#### 4.14.5. Analysis of EHMC in Rat Plasma

Plasma samples were thawed on ice, and 0.2 mL aliquots were spiked with an internal standard. Acetonitrile (three aliquots of threefold volume, 3 × 0.2 mL) was inserted sequentially, with vortexing for 5 s before and after each addition. The mixture was centrifuged at 12,000× *g* for 20 min, and the supernatant was harvested for analysis using a high-performance liquid chromatography-triple quadrupole mass spectrometry system (LC-MS 8060, Shimadzu, Kyoto, Japan).

Analytical Conditions:

(i) Liquid Chromatography (LC) Conditions: The mobile phase consisted of 75% acetonitrile/water (*v*/*v*) containing 0.1% formic acid. The analysis was carried out using a C18 column (1.2 mm × 50 mm, 5 μm particle size) at a flow rate of 0.4 mL/min and a column temperature of 40 °C.
(ii) Mass Spectrometric (MS) Conditions:

Analysis was undertaken in multiple reaction monitoring (MRM) mode. The dwell time for each transition was set to 100 ms. Precursor ions, product ions, and collision energies (CE) were optimized as detailed in Table 14.

### 4.15. Statistical Analysis

Data are presented as mean ± standard deviation (SD) where replicate-level data were available. Statistical analysis was performed using GraphPad Prism 10.1.2 software (GraphPad Software, San Diego, CA, USA). For multi-group comparisons, one-way analysis of variance (ANOVA) followed by Dunnett’s post hoc test was used. A value of *p* < 0.05 was considered statistically significant. For endpoints reported without replicate-level variance, the data were interpreted descriptively and used only for comparative trend analysis.

## Figures and Tables

**Table 1:** Experimental results of drug loading and SPF values in the RSM design.

NO	Drug Loading (%)	SPF
1	23.825	2.53
2	39.004	3.13
3	18.691	3.13
4	27.836	3.17
5	26.15	3.03
6	41.016	3.00
7	28.185	1.99
8	34.035	3.19
9	46.631	2.17
10	24.081	2.66
11	32.276	2.31
12	31.041	2.04
13	44.439	4.33
14	45.223	4.19
15	46.554	4.17
16	46.564	4.29
17	46.689	4.3

**Table 2:** Variance analysis for the established regression model.

Source	Drug Loading (%)			SPF		
	F-Value	*p*-Value	Sig.	F-Value	*p*-Value	Sig.
Model	85.44	<0.0001	**	90.03	<0.0001	**
A	131.87	<0.0001	**	30.30	0.0009	**
B	104.46	<0.0001	**	6.84	0.0346	*
C	9.90	0.0162	*	16.36	0.0049	**
AB	4.73	0.0661	ns	5.80	0.0469	*
AC	10.57	0.0140	*	27.99	0.0011	**
BC	59.07	0.0001	**	10.69	0.0137	*
A2	212.79	<0.0001	**	44.81	0.0003	**
B2	165.63	<0.0001	**	245.00	<0.0001	**
C2	29.81	0.0009	**	359.56	<0.0001	**
Missing item	3.06	0.1540	ns	4.87	0.0801	ns

Note: *p* < 0.01 indicates extremely significant effect (**); *p* < 0.05 indicates significant effect (*); ns indicates no significant effect.

**Table 3:** Analysis of data of response model.

Response Value Metric	Mean	Standard Deviation	R^2^	R^2^_adj_	R^2^_pre_	C.V.%	Adequate Precision
Drug Loading	35.43	1.39	0.9910	0.9794	0.8952	3.91	26.0332
SPF	3.15	0.1162	0.9914	0.9804	0.8895	3.68	24.7846

**Table 4:** In vitro SPF values of tested formulation.

Sample	Agents	First Test	Second Test	Third Test	Average Values
SC-1	10 wt% EHMC	15.13	14.42	13.32	14.29
SC-2	10 wt% EHMC + 4 wt% DDH	16.98	15.92	17.98	16.96
SC-4	10 wt% EHMC + 4 wt% DDH + 1 wt% DP	15.42	15.11	14.34	14.96
RSM-Optimal	6.5 wt% EHMC + 4 wt% DDH + 3 wt% DP	13.18	13.97	13.82	13.66

**Table 5:** In vitro SPF values of application formulations.

Sample	Agents	First Test	Second Test	Third Test	Average Values
Base cream	None	10.44	9.58	11.22	10.41
SC-1	10 wt% EHMC	15.30	18.50	16.37	16.72
SC-2	10 wt% EHMC + 4 wt% DDH	25.80	22.40	21.15	23.11 *
SC-3	10 wt% EHMC + 1 wt% DP	19.80	17.30	19.17	18.75
SC-4	10 wt% EHMC + 4 wt% DDH + 1 wt% DP	27.70	22.50	22.17	24.12 *

Note: * *p* < 0.05 compared with the SC-1 group.

**Table 6:** Particle Size Measurements for Samples SC-2 and SC-4.

Formulation Number	D10	D10 Average Value	D50	D50 Average Value	D90	D90 Average Value
SC-2	121.4 μm	123.2 μm	155.7 μm	160.3 μm	202.4 μm	207.1 μm
	123.3 μm		160.6 μm		207.8 μm	
	124.9 μm		164.6 μm		211.1 μm	
SC-4	0.456 μm	0.454 μm	3.423 μm	3.894 μm	109.3 μm	114.8 μm
	0.453 μm		3.840 μm		116.6 μm	
	0.454 μm		4.418 μm		118.4 μm	

**Table 7:** Zeta Potential Measurements for Samples SC-2 and SC-4.

Formulation Number	Zeta Potential	Zeta Potential Average Value
SC-2	8.98 mV	9.28 mV
	9.31 mV	
	9.55 mV	
SC-4	−15.20 mV	−14.80 mV
	−15.10 mV	
	−14.10 mV	

**Table 8:** Viscosity Changes in the SC-2 Film-Forming Formulation Samples During Temperature Stability Assay.

Time	Temperature			
	−15 °C	4 °C	25 °C	45 °C
Day 0	9800 mPa·S	9800 mPa·S	9800 mPa·S	9800 mPa·S
Day 7	8165 mPa·S	8525 mPa·S	8600 mPa·S	8141 mPa·S
Day 14	6480 mPa·S	8080 mPa·S	8600 mPa·S	8840 mPa·S

**Table 9:** Viscosity Characteristics of the SC-4 Film-Forming Formulation Samples During Temperature Stability Assay.

Time	Temperature			
	−15 °C	4 °C	25 °C	45 °C
Day 0	6720 mPa·S	6720 mPa·S	6720 mPa·S	6720 mPa·S
Day 7	6500 mPa·S	6853 mPa·S	6400 mPa·S	6231 mPa·S
Day 14	6320 mPa·S	6360 mPa·S	6240 mPa·S	6080 mPa·S

**Table 10:** Pharmacokinetic parameters of EHMC following topical application in rats.

Group	Individual C_max_ (ng/mL)	Individual T_max_ Median (Range), h	AUC_0–48h_ (ng·h/mL)	AUC CV (%)
SC-1	2.287 ± 0.357	2 (1–4)	64.130 ± 10.636	16.6
SC-2	3.184 ± 1.240	1 (1–2)	80.688 ± 38.127	47.3
SC-4	2.295 ± 0.540	8 (2–48)	60.605 ± 9.405	15.5

**Table 11:** Experimental design of the response surface methodology.

NO	Factor		
	A EHMC	B DDH	C DP
1	−1 (4)	−1 (2)	0 (3)
2	+1 (8)	−1 (2)	0 (3)
3	−1 (4)	+1 (6)	0 (3)
4	+1 (8)	+1 (6)	0 (3)
5	−1 (4)	0 (4)	−1 (1)
6	+1 (8)	0 (4)	−1 (1)
7	−1 (4)	0 (4)	+1 (5)
8	+1 (8)	0 (4)	+1 (5)
9	0 (6)	−1 (2)	−1 (1)
10	0 (6)	+1 (6)	−1 (1)
11	0 (6)	−1 (2)	+1 (5)
12	0 (6)	+1 (6)	+1 (5)
13	0 (6)	0 (4)	0 (3)
14	0 (6)	0 (4)	0 (3)
15	0 (6)	0 (4)	0 (3)
16	0 (6)	0 (4)	0 (3)
17	0 (6)	0 (4)	0 (3)

**Table 12:** Response surface test factor level table.

Level	Factor		
	A EHMC (%)	B DDH (%)	C DP (%)
−1	4	2	1
0	6	4	3
1	8	6	5

**Table 13:** Composition of the final application formulation set used for SPF, film-formation, stability, and in vivo evaluations.

Phase Types	INCI	Formulation Number				
		Base Cream	SC-1	SC-2	SC-3	SC-4
Aqueous Phase	Deionized Water	14.10	14.10	14.10	14.10	14.10
	Butylene Glycol	8.00	8.00	8.00	8.00	8.00
	Sodium Chloride	0.80	0.80	0.80	0.80	0.80
	90% Phenoxyethanol and Ethylhexylglycerin (9010)	0.60	0.60	0.60	0.60	0.60
Oil Phase	Ethylhexyl Methoxycinnamate (EHMC)	0.00	10.00	10.00	10.00	10.00
	Diisopropyl Sebacate	10.00	10.00	10.00	10.00	10.00
	Dextrin Palmitate	0.00	0.00	0.00	1.00	1.00
	P135	1.50	1.50	1.50	1.50	1.50
	Cetyl PEG/PPG-10/1 Dimethicone	1.00	1.00	1.00	1.00	1.00
	C12-15Alkyl Benzoate	30.00	30.00	30.00	30.00	30.00
	Disteardimonium Hectorite	0.00	0.00	4.00	0.00	4.00
	VP/Eicosene Copolymer (VP)	4.00	4.00	4.00	4.00	4.00
	Cyclopentasiloxane	30.00	20.00	16.00	19.00	15.00
Summation of Formulation Component Proportions		100.00	100.00	100.00	100.00	100.00

Note: The RSM-optimal formulation (6.5 wt% EHMC, 4 wt% DDH, and 3 wt% DP) was prepared separately for model validation and is not included in this table unless otherwise specified.

**Table 14:** Precursor ions, product ions, and collision energies (CE).

Analyte	Precursor Ion (*m*/*z*)	Product Ion (*m*/*z*)	Collision Energy (eV)
Ethylhexyl Methoxycinnamate	291.20	161.10	−20
		179.10	−9
		133.10	−30

## Data Availability

The original contributions presented in this study are included in the article. Further inquiries can be directed to the corresponding authors.

## Abbreviations

The following abbreviations are used in this manuscript:

4-MBA	4-methoxybenzaldehyde
ANOVA	analysis of variance
DDH	disteardimonium hectorite
DP	dextrin palmitate
DSC	differential scanning calorimetry
EHMC	2-ethylhexyl-4-methoxycinnamate
FT-IR	Fourier-transform infrared
H&E	hematoxylin and eosin
HPLC	high-performance liquid chromatography
LC-MS	liquid chromatography–mass spectrometry
MSI	mass spectrometry imaging
PBS	phosphate-buffered saline
PDMS	polydimethylsiloxane
PFA	paraformaldehyde
PMMA	poly (methyl methacrylate)
RSM	response surface methodology
SC	sunscreen
SEM	scanning electron microscopy
UVA-PF	UVA protection factor
SNR	signal-to-noise ratio
SPF	sun protection factor
TEWL	transepidermal water loss
TGA	thermogravimetric analysis
TiO_2_	titanium dioxide
UV	ultraviolet
VP	vinylpyrrolidone
ZnO	zinc oxide
